# Effects of the New Drug Law

**Published:** 1849-03

**Authors:** 


*Effects of the New Drug Law.*—Dr. Edwards, the indefatigable chairman of the Special Committee in Congress, which reported the new law, was employed for some time before the commencement of the present session, by Mr. Secretary Walker, to visit the ports of New York, Philadelphia, Baltimore and Boston, and report upon the operation of the law. Through the kindness of Dr. Bœrstler, of Lancaster, we have been favored with a few items from Dr. Edwards' forthcoming report. We are sure we could not lay more highly interesting and satisfactory information before our readers, than is contained in the following extracts from Dr. Bœrstler's letter to us.

"Much good has already resulted from the law, and the opposition to it is now confined to Drug brokers, the regular dealers approbating it. The partner of the largest importing house in New York, now in Europe, writes that this law will effectually prevent the shipment of adulterated drugs and chemicals. The inspectors of our ports are doing their duty faithfully and fearlessly—instance New York alone, where the following spurious articles were not allowed to enter, but sent back. The inspector rejected:

| | | | | | |
|---|---|---|---|---|---|
| July 19, | 6,650 | lbs. | spurious rhubarb root, | from | Canton. |
| " 21, | 931 | " | " " " | " | " |
| Aug. 10, | 750 | " | adulterated opium, | " | Marseilles. |
| " 11, | 2,940 | " | jalap root, spurious, | " | Tampico. |
| " 31, | 2,259 | " | rhubarb root, spurious, | " | London. |
| Sep. 1, | 645 | " | " " " | " | " |
| " 5, | 1,414 | " | gamboge, | " | " |
| " 8, | 545 | " | rhubarb root, | " | Hamburg. |
| " 9, | 1,400 | " | senna, | " | Leghorn. |
| " 19, | 2,930 | " | yellow bark, spurious, | " | Bordeaux. |
| " 20, | 875 | " | rhubarb root, | " | Canton. |
| " 22, | 758 | " | opium, | " | London. |
| " 25, | 1,783 | oz. | iodine, | " | " |
| " 26, | 1,075 | lbs. | rhubarb root, | " | Marseilles. |
| " 26, | 875 | " | jalap root, | " | Vera Cruz. |
| " 29, | 3,100 | " | " | " | " |
| Oct. 23, | 788 | " | rhubarb, | " | London. |
| " 23, | 227 | " | gum myrrh, | " | " |
| " 25, | 13,120 | " | yellow bark, spurious, | " | Marseilles. |
| " 26, | 1,875 | " | " " " | " | Bordeaux. |
| " 26, | 412 | " | gum myrrh, | " | London. |

In Boston a number of cases of adulterated opium, and other spurious drugs were also rejected. This will give your readers some little idea of the rascality of traders in such things, and shows the necessity of penal legislation. As well might men poison our fountains as sell us adulterated medicinal drugs, &c. * * * * *

It was apprehended the revenue would suffer by this law, but Mr. Secretary Walker nobly said, "let no revenue be derived from any nefarious traffic." So far from injury, the revenue is increasing, because genuine drugs and chemicals are taking the place of spurious—for example, the spurious yellow bark was invoiced at $4 per 100 lbs. when the genuine is worth $70 to $90 per 100 lbs. So in other articles.—*Ohio Med. and Surg. Journal.*

